# Persistence of Cytosine Methylation of DNA following Fertilisation in the Mouse

**DOI:** 10.1371/journal.pone.0030687

**Published:** 2012-01-26

**Authors:** Yan Li, Chris O'Neill

**Affiliations:** Sydney Medical School, Centre for Developmental and Regenerative Medicine, Kolling Institute for Medical Research, University of Sydney, Sydney, Australia; UCLA-DOE Institute for Genomics and Proteomics, United States of America

## Abstract

Normal development of the mammalian embryo requires epigenetic reprogramming of the genome. The level of cytosine methylation of CpG-rich (5meC) regions of the genome is a major epigenetic regulator and active global demethylation of 5meC throughout the genome is reported to occur within the first cell-cycle following fertilization. An enzyme or mechanism capable of catalysing such rapid global demethylation has not been identified. The mouse is a widely used model for studying developmental epigenetics. We have reassessed the evidence for this phenomenon of genome-wide demethylation following fertilisation in the mouse. We found when using conventional methods of immunolocalization that 5meC showed a progressive acid-resistant antigenic masking during zygotic maturation which gave the appearance of demethylation. Changing the unmasking strategy by also performing tryptic digestion revealed a persistence of a methylated state. Analysis of methyl binding domain 1 protein (MBD1) binding confirmed that the genome remained methylated following fertilisation. The maintenance of this methylated state over the first several cell-cycles required the actions of DNA methyltransferase activity. The study shows that any 5meC remodelling that occurs during early development is not explained by a global active loss of 5meC staining during the cleavage stage of development and global loss of methylation following fertilization is not a major component of epigenetic reprogramming in the mouse zygote.

## Introduction

The dominant paradigm describing the processes of epigenetic reprogramming in the embryo holds that global active demethylation of 5meC occurs within the first cell-cycle. This demethylation acts preferentially on DNA inherited from the male while passive demethylation of the maternally derived genome occurs over subsequent mitoses [1]. The mouse is a widely used model for studying developmental epigenetics. The DNA that the fertilized mouse embryo inherits from gametes has relatively low levels of 5′-methylation of CpG (5meC) rich regions. By the blastocyst stage (∼80 cells) this level shows some further reduction prior to a round of *de novo* methylation as the inner cell mass forms the epiblast. The mechanism that is currently considered to best describe 5meC reprogramming should result in an almost complete loss of methylation (<1%) by the time the embryo reaches the blastocyst stage. Analysis of around 1000 CpG islands (CGIs) within ovulated eggs shows that 15% are methylated [2]. The level is higher (∼25%) in sperm but the proportion of individual CpGs methylated in CGIs in sperm is lower [2]. By the blastocyst stage many of these methylated CGIs show some loss of methylation but not to the very low levels predicted by the accepted model for epigenetic reprogramming [1]. Furthermore, a significant minority of non-imprinted methylated CGIs in gametes remained hypermethylated in blastocysts. Only a relatively small number of CGIs showed substantial demethylation [2]. This higher than expected level of methylation in blastocysts might be accounted for by substantial remethylation after post-fertilization demethylation, yet MeDIP analysis shows that the major round of *de novo* methylation occurs later, upon epiblast formation (D6.5)[3].

Reports of an active process of global 5meC demethylation of the zygotic genome within hours of fertilisation in some species (mouse, rat, bovine [4], [5], [6]) have prompted an extensive but so far unsuccessful search for a mammalian CpG demethylase capable of catalysing this feat [7], [8]. In other species such global demethylation was not consistently observed (sheep [9], [10], rabbit [11]) and the evidence for active demethylation is equivocal for other species (human [12], pig [13]). Furthermore, there is some evidence that global demethylation immediately following fertilisation is not required for successful embryo development [14].

In this study we undertook a systematic reanalysis of global 5′-methylated CpG levels in the fertilised zygote by a conventional immunolocalization approach and by an alterative method of detecting the binding of the selective 5meC binding protein, methyl binding domain 1 protein (MBD1). This re-analysis did not find evidence for extensive active loss of methylation in zygotes or progressive loss due to an absence of maintenance methylation across the first several rounds of cell division. Rather, it was found that the reported loss of methylation immediately after fertilisation was accounted for by changes in the conformation or structure of chromatin that resulted in antigenic masking of 5meC.

## Results

**Figure 1:**
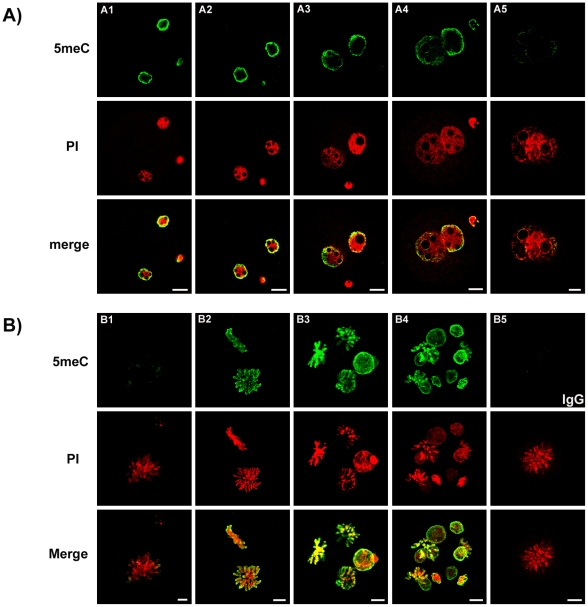
Pattern of anti-5meC staining in early preimplantation stage embryos. Zygotes were collected directly from the oviduct 16 h – 25 h after the ovulatory injection of hCG. Images show zygotes at PN stage 1-5 (**A1-5**) and condensing chromosomes (**B1**), 2-cell (**B2**), 4-cell (**B3**) and 8-cell (**B4**). Mitotic zygotes stained with non-immune IgG control is also shown (**B5).** Antigenic unmasking of 5meC (green) was by brief acid exposure. DNA was counter stained with propidium iodide (PI, Red). Images from these two channels were merged to show co-localization (merge). Images of zygotes were single-equatorial confocal sections (0.77 µm), images of nuclei from 2-cell to 8-cell stage were Z-stacks of multiple sections through each nuclei of the embryo. The images shown here are representative of at least seven independent replicate experiments with at least 10 embryos per observation group per replicated. The scale bars are 10 µm.

**Figure 2:**
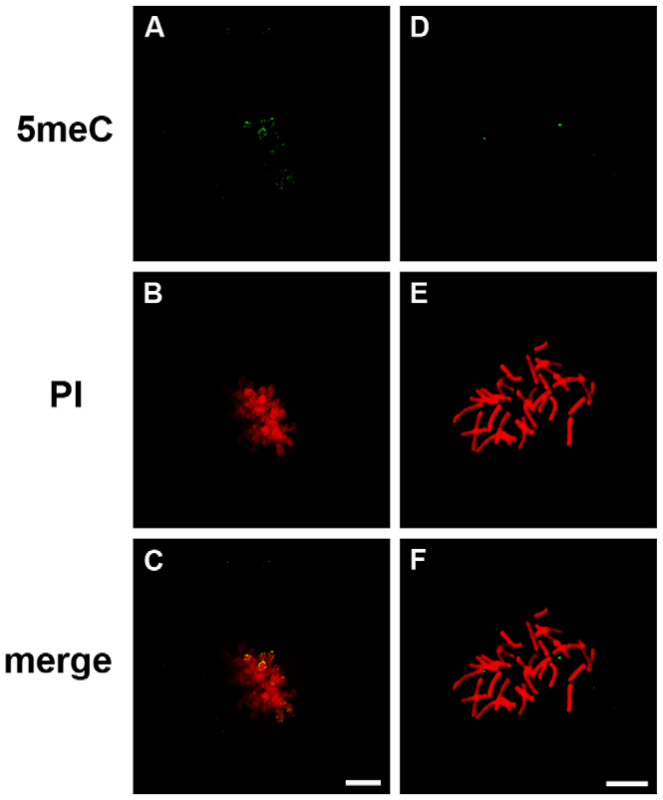
Effect of different methods of fixation on 5meC staining. Condensed chromosomes from zygotes (**A-C)** were fixed as in Figure 1 or were subjected to the air-dried and methanol fixation method (D-F). The resultant chromosomes from both methods failed to display any significant decoration by anti-5meC. The images show the anti-5meC (5meC), propidium (PI) staining and the merged imaged of these two channels (merge). The images shown here are representative of at least three independent replicate experiments with at least 10 embryos per observation group per replicated. The scale bars are 10 µm.

Mouse zygotes, 2-cell, 4-cell and 8-cell embryos were collected directly from the female reproductive tract from B6CBF1 strain female mice (mated with males of the some strain). The embryos were fixed and immunostained with anti-5meC. Zygotes were collected at various times after mating and staged according to the maturation of their pronuclei (PN1 being least and PN5 most mature [15]). This analysis revealed anti-5meC staining of both the male and female pronuclei in the PN1 (Fig. 1A1) and PN2 stages (Fig. 1A2), a progressive reduction in the relative intensity of anti-5meC staining at the PN3 (Fig 1A3) and PN4 (Fig 1A4) stages but little detectable anti-5meC staining in either the maternal or paternal pronuclei was observed in the PN5 stage zygote (Fig 1A5). Further analysis showed no detectable anti-5meC staining on condensing chromosomes in zygotes (Fig 1B1), yet condensing chromosomes from the 2-cell embryo (52 h post-hCG) were heavily decorated with anti-5meC (Fig 1B2). Both the interphase nuclei and condensing chromosomes in 4-cell (Fig 1B3) and 8-cell (Fig 1B4) embryos all showed a consistent pattern of nuclear anti-5meC staining. These patterns of staining were consistent over many independent replicates using a range of different microscope systems. Non-immune IgG caused no staining of metaphase chromosomes (Fig 1B5). The absence of staining by anti-5meC in zygotic chromosomes was unlikely to be a fixation artefact since zygotic chromosomes showed similar decoration whether the zygotes were conventionally fixed (Fig 2A–C) or fixed by an alternative air-dried method [16] (Fig 2D–F).

**Figure 3:**
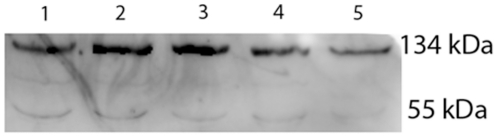
MBD1 expression in embryos. Western blot of MBD1 antigen was performed for oocytes and embryos at various stages of preimplantation development (1 – ovulated oocytes; 2 – zygotes; 3 – 2-cell; 4 - 8-cell; 5 – blastocyst). Representative of three independent replicates.

**Figure 4:**
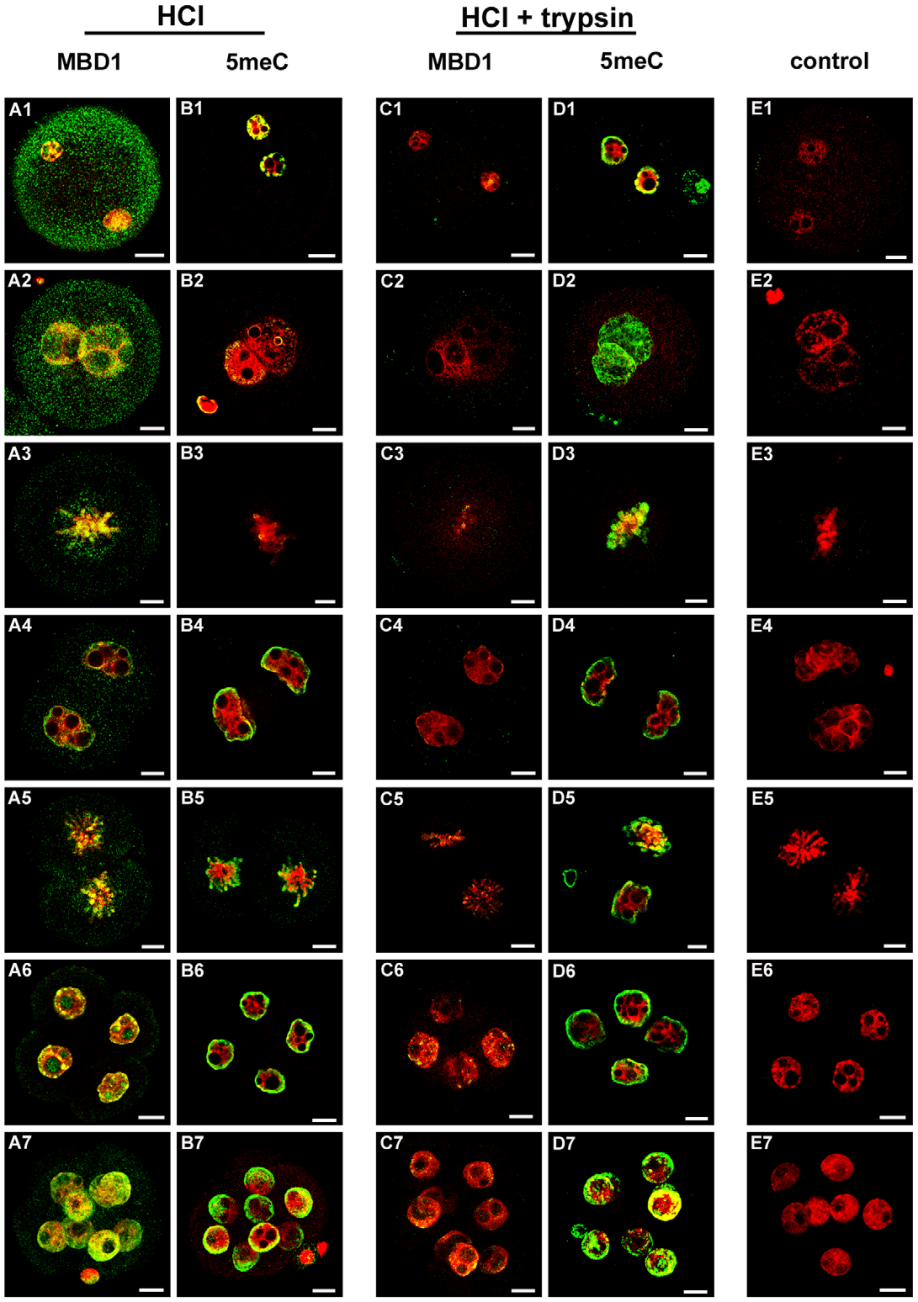
Localization of 5meC in the early embryo by staining for MBD1 and the effect of tryptic digestion on antigenic unmasking of 5meC. Embryos were freshly collected from the reproductive tract and fixed and antigen unmasked (HCl) as in Fig 1. They were then stained with either anti-MBD1 (MBD1) (A and C ), anti-5meC (5meC) (B and D), non-immune IgG (E) (control). Some embryos were subjected to further antigenic unmasking by tryptic digestion (HCl + trypsin) (C, D, E). Embryos were assessed at the (1) zygotic PN2, (2) PN5, (3) zygotic metaphase, (4) interphase 2-cell, (5) 2-cell metaphase, (6) 4-cell, and (7) 8-cell stages. Each image is a single (0.77 µm) confocal section through embryos except metaphase 2-cell chromosomes and 8-cell embryos which are complied z-stacks of multiple sections. The images shown here are representative for at least seven independent replicate experiments with at least 10 embryos per observation group per replicated. The scale bars are 10 µm.

Detection of a class of methylcytosine-binding proteins, typified here by methyl binding domain 1 protein (MBD1), is an independent measure of the global levels of CpG methylation. Western blot analysis showed two dominant molecular weight forms of MBD1 present in oocytes and the early stages of development (Fig 3). Immunolocalization of MBD1 with this same antibody showed that it was present in each cell of the developing embryo, and that it showed considerable accumulation within nuclei. In the zygote, this staining was present in PN2 (Fig 4A1) through to the PN5 (Fig 4A2) stages and also in the condensing chromosomes of zygotes (Fig 4A3). This MBD1 staining was present in the 2-cell interphase nuclei (Fig 4A4) and persisted in the 2-cell condensing chromosomes (Fig 4A5) and in the 4-cell to 8-cell interphase nuclei (Fig 4A6 & 7). Some embryos from each of these cohorts were stained with the anti-5meC antibody (Fig 4B1–7), and this confirmed the loss of anti-5meC staining in the late stage zygotes (Fig 4B2, 3). Thus, anti-MBD1 and anti-5meC gave a similar measure of global 5meC at each stage of early development tested, except during the late stages of zygotic maturation.

**Figure 5:**
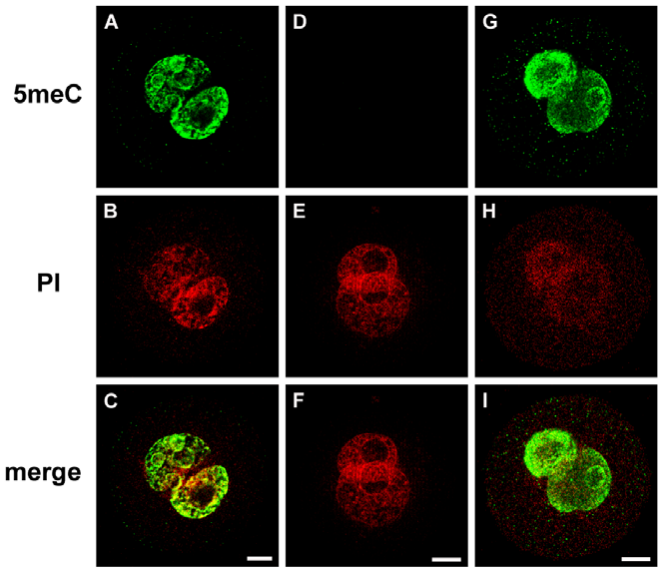
Specificity of 5meC staining. Zygotes at the PN5 stage were antigenically unmasked by combined acid and trypsin pretreatment and stained with anti-5meC as described in Fig 1, (**A-C**) or stained with anti-5meC in the presence of excess (0.6 µM ) free 5meC (Sigma) (**D-F**). To further asses specificity staining with an anti-5meC from an alternative source was performed (**G-I**). (mouse monoclonal to 5meC, used as 1∶100 dilution and incubated at 4°C for 18 h; Abcam ab73938). This showed the same pattern of staining as was the antibody used in the rest of the study. Representative of three independent replicates.

Brief acid-treatment of cells has been the most widely used method of 5meC antigen retrieval reported. Altering the duration and concentration of HCl treatment did not change the pattern of 5meC staining in PN5 zygotes (not shown) showing that the difference between anti-5meC and anti-MBD1 staining was not due to increased acid-sensitive masking of 5meC. To determine whether other forms of masking may have occurred, fixed and acid-treated zygotes were subjected to extensive tryptic digestion. This caused a loss of MBD1 staining from nuclei (Fig 4C1–7) and a concomitant increase in the staining by anti-5meC in the PN5 zygotes. The level of 5meC staining in trypsin treated embryos remained similar at all stages of zygotic maturation (Fig 4D1–7) including the PN5 pronuclei (Fig 4D2) and zygotic chromosomes (Fig 4D3). Non-immune serum did not result in any staining of embryos subjected to tryptic digestion (Fig 4E1–7). After tryptic digestion anti-5meC staining (Fig 4 D1–7) revealed a pattern of global cytosine methylation that was similar to that revealed by MBD1 binding in the absence of trypsin treatment (Fig 4A1–7). This staining pattern was consistently observed in many independent replicates. Anti-5meC staining (Fig 5A–C) was blocked by the presence of excess free 5meC antigen (Fig 5D–F) and a similar pattern of anti-5meC staining was observed upon use of an alternative antibody (Fig 5G–I). Staining with a sheep polyclonal anti-5meC (AbD Serotec, AHP1826z) also showed similar patterns of staining (not shown). The results show that the loss of anti-5meC staining during zygotic maturation is due to the onset of acid-resistant but trypsin-sensitive antigenic masking of 5meC.

**Figure 6:**
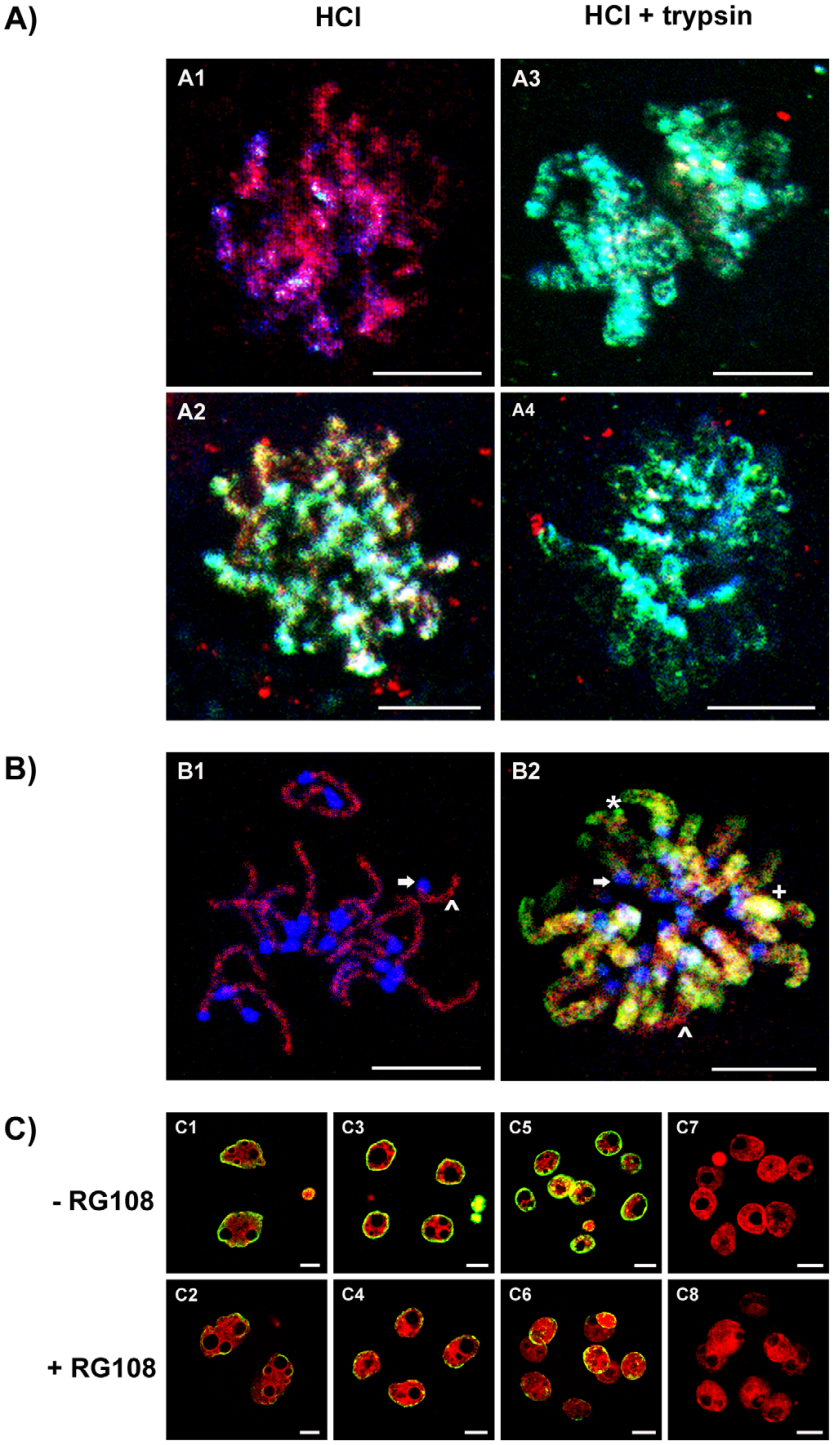
Relative distribution of MBD1 and 5meC staining on metaphase chromosomes. **A**) Triple staining of MBD1, 5meC and DNA in 1-cell and 2-cell metaphase chromosomes after antigenic unmasking by acid or acid plus trypsin. Zygotes (**A1** and **A3**) and 2-cell (**A2** and **A4**) embryos were collected at 31 and 53h after hCG, respectively, as they were entering metaphase. They were fixed and treated with acid alone (**A1** and **A2**) or acid followed by acid plus trypsin (HCl + trypsin) (**A3** and **A4**). DNA was counter-stained with DAPI (purple), anti-5meC (FITC, green) and MBD1 (red). Images shown are the merged result of these three channels. Regions of DNA in which anti-5meC and anti-MBD1 are co-localised appear white-yellow, regions of anti-MBD1 alone are pink, and those with anti-5meC alone stain blue-green. Images are z-stacks of multiple confocal sections through the chromosomes. Representative of three independent replicates. The scale bars are 10 µm. **B**) High resolution image of triple-stained metaphase chromosomes from zygotes and 2-cell embryos. Condensed chromosomes from zygotes (**B1**) were fixed by the air-dried method [16] and 2-cell condensed chromosomes (**B2**) by formaldehyde. Images were captured using a 100× oil objective and multiple confocal sections through the chromosomes were collected and the Z-stack images compiled into a two-dimensional representation. Regions of the genome are illustrated as follows: ↑, undecorated DNA; ∧, DNA stained only with anti-MBD1; *, DNA stained only with anti-5MeC; +, DNA dual stained with anti MBD1 and anti-5meC. Representative of three independent replicates. The scale bars are 10 um. **C**)The role of DNA methyltransferase in the maintenance of 5meC over the first cell cycles. Zygotes (25h post-hCG) were cultured in standard media [35] (- RG108) or media supplemented with RG108 (5 µM) for 16 h. This incubation period covered the time of DNA synthesis in the 2-cell embryo [37]. The embryos were then fixed and stained with anti-5meC (control – **C1**, RG108 – **C2**) or the embryos were extensively washed and then cultured for another 24 h (control – **C3**, RG108 – **C4**) or 32 h (control – **C5**, RG108 – **C6**) prior to staining with anti-5meC. Non-immune controls (control – **C7**, RG108 – **C8**). Representative of five independent replicates with at least 10 embryos per treatment dose. The scale bars are 10 µm.

To further assess this antigenic masking, condensing chromosomes of the zygote and 2-cell embryo were triple stained for DNA (blue), 5meC (green) and MBD1 (red). Acid-treated chromosomes from the zygote had some segments of undecorated DNA and large segments decorated by MBD1, but were largely devoid of staining by anti-5meC (Fig 6A1). By contrast, similarly treated chromosomes from 2-cell embryos had large segments that were co-decorated by both anti-5meC and anti-MBD1 (Fig 6A2). Treatment of zygotes with trypsin removed most MBD1 staining from chromosomes and this revealed large regions stained with anti-5meC (Fig 6A3). Two-cell chromosomes treated in this manner lost most MBD1 staining but this did not result in a marked change the pattern of anti-5meC staining (Fig 6A4). Given the apparent regionalisation of staining of anti-MBD1 and anti-5meC, acid-treated chromosomes were examined at higher resolution (Fig 6B). This confirmed the trypsin-sensitive exclusion of anti-5meC from zygotic chromosomes, but revealed large segments of each chromosome that remained methylated and bound MBD1 (Fig 6B1). The condensing chromosomes of the 2-cell embryo (Fig 6B2) displayed a different conformation; with large segments being methylated, as shown by binding of both anti-5meC and anti-MBD1; only small segments showed anti-MBD1 staining in the absence of anti-5meC (the same pattern as zygotes); and small segments showing predominantly anti-5meC staining in the absence of MBD1 was observed. The differences in anti-5meC staining pattern and changes in acid-resistant masking of 5meC between zygotes and 2-cell embryos reveals a remarkable change in the conformation and/or structure of chromatin over these first two cell-cycles.

It is reported that further passive demethylation of the embryonic genome occurs during the cell-cycles following fertilisation due to limited maintenance methylation of the newly synthesized DNA [1], [17]. Failure of maintenance methylation would be expected to result in a ∼90% reduction in the level of global methylation by the 8-cell stage. Our analysis (Figs 1 & 4) did not reveal a reduction in global 5meC staining from the 2-cell to 8-cell stages. The role of DNA methyltransferases in this persistence in staining over the first cell-cycles was examined by use of the selective DNA methyltransferase inhibitor, RG108. Zygotes were collected at the PN5 stage and cultured in the presence of RG108 until the completion of DNA synthesis in the second cell-cycle. Compared to culture in control media (Fig 6C1), RG108 caused a marked loss of 5meC staining in the resulting G2-phase 2-cell embryos (Fig 6C2). Some embryos were washed extensively to remove RG108 and then cultured to the 4-cell or 8-cell stage followed by staining for 5meC. Embryos cultured to the 4-cell stage without exposure to RG108 had similar levels of anti-5meC staining in each nuclei (Fig 6B3) as untreated 2-cells, but the level of anti-5meC staining in embryos exposed to RG108 during the 2-cell stage and then cultured in the absence of inhibitors remained at a low level (Fig 6C4). A similar level of staining was observed in 8-cell embryos (Fig 6C5,6). Non-immune serum did not result in staining (Fig 6C7,8). The lower level of staining in RG108-treated embryos after one cell division shows that the 5meC immunolocalization method had ample sensitivity to detect a 50% reduction in global methylation. There was no evidence for a progressive loss of methylation with each cell division and certainly no evidence for a 90% loss of methylation at the 8-cell stage in untreated embryos as is predicted by the current model of epigenetic reprogramming. The persistence of 5meC in the nuclei of untreated 2-cell, 4-cell and 8-cell embryos does not support a role for global passive demethylation of the genome as a major mechanism associated with the normal development of the early embryo. A RG108-sensitive DNA methyltransferase activity during the 2-cell S-phase was required for maintenance of normal levels of 5meC staining in early preimplantation embryo. This result is consistent with recent findings of the presence and action of DNA methyltransferase 1 (DNMT1) in the early stage embryo [18],[19].

**Figure 7:**
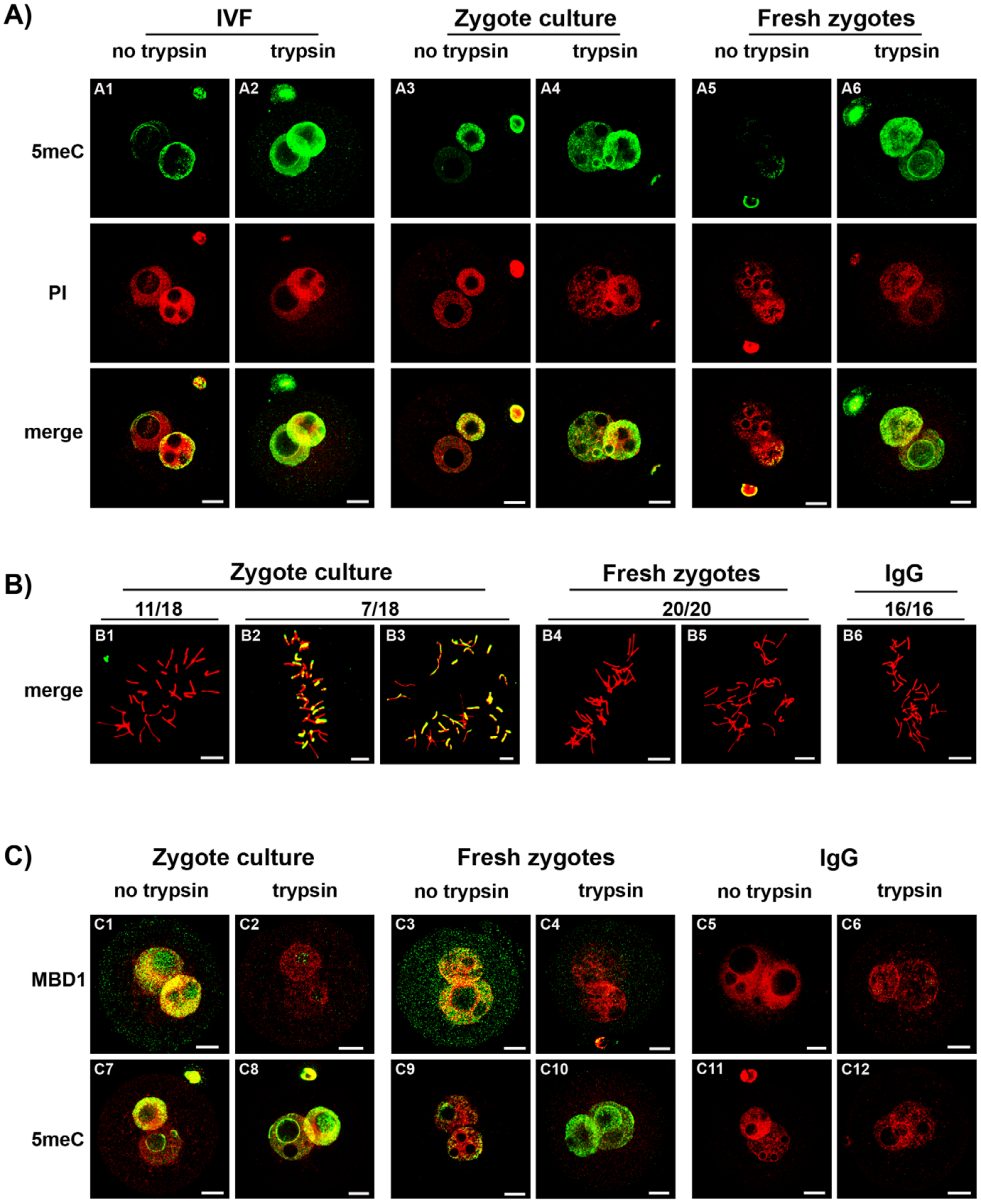
The effect of embryo manipulation on 5meC staining in zygotes. **A**)Zygotes were created by routine mouse IVF [40] and culture in vitro (8 h) to the PN5 stage (IVF) (**A1** and **A2**) ; collected after fertilization in the reproductive tract 17 h after hCG and then culture in vitro for 8 h to the PN5 stage (Zygote culture) (**A3** and **A4**), or they were collected directly from the reproductive tract at the PN5 stage, and fixed without further treatment (Fresh zygotes) (**A5** and **A6**). The zygotes were fixed, subjected to acid unmasking and then either buffer (no trypsin) (**A1**, **A3** and **A5**) or tryptic digestion (trypsin) (**A2**, **A4** and **A6**) as in Fig 2. Zygotes were stained with anti-5meC (green) or PI (red) and both channels merged. Representative of three independent replicates. The scale bars are 10 µm. Zygotes were prepared as in (A) but were fixed with methanol by the air-dried method [16] and stained during chromosome condensation. In cultured zygotes a proportion showed variable segments of chromosome that stained for 5meC (**B1-3**). In Fresh zygotes no 5meC staining was observed (**B4**-**5**). The proportion of the total number of metaphase zygotes from each treatment that showed the illustrated pattern of staining is shown above the figures. No 5meC staining was observed in no-immune control (**B6**). The scale bars are 10 µm.

**Figure 8:**
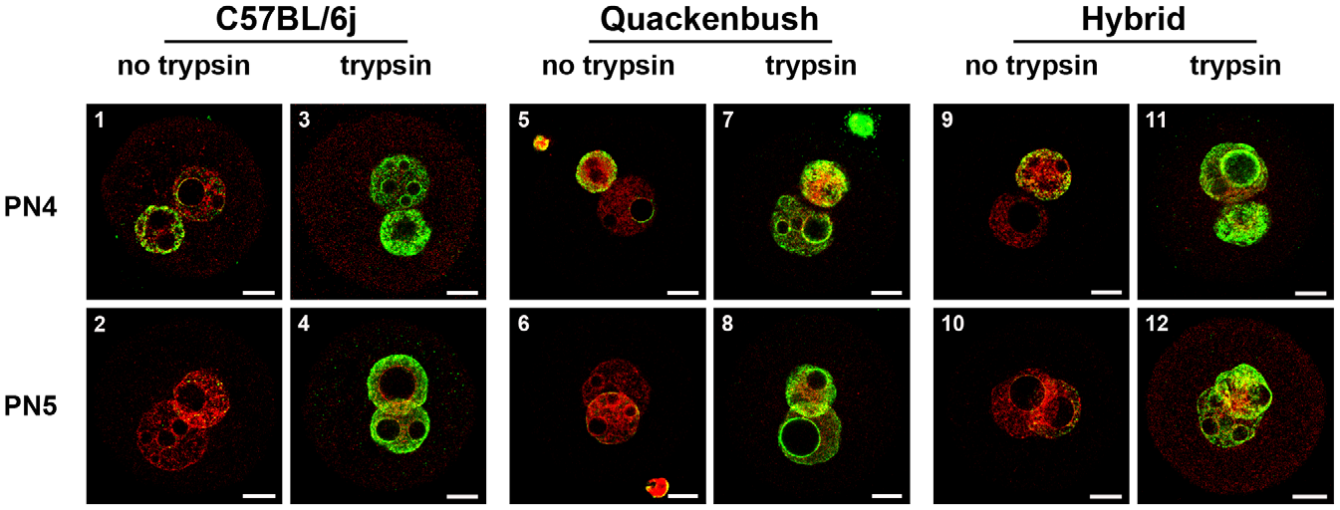
Effect of antigen masking of 5meC in three different mouse strains. PN4 and PN5 stage zygotes were collected from inbred (C57BL/6j), outbred (Quackenbush) and B6CBF1 (hybrid) females mated with the same strain male. Embryos were fixed and unmasked with acid alone (no trypsin, 1,2,5,6,9,10) or acid and trypsin (trypsin, 3,4,7,8,11,12) and stained with anti-5meC. The results are representative of three independent replicates.

Asymmetric anti-5meC staining of the male and female pronucleus after acid-pretreatment has been reported [5], [6], [20], [21] yet was not confirmed by this study. The zygotes used in past studies were commonly generated by in vitro fertilization or subjected to culture in vitro (which provides logistic advantages for the feasibility of such studies). We repeated the analysis using embryos generated by in vitro fertilization or collected soon after fertilization and then cultured in vitro. After antigenic unmasking with acid, the smaller (female) pronucleus in zygotes produced by in vitro fertilization (Fig 7A1) and or cultured in vitro (Fig 7A3) showed more anti-5meC staining compared to those collected directly from the oviduct (Fig 7A5). After antigenic unmasking by acid and trypsin, however, high levels of anti-5meC staining were consistently observed in both pronuclei of IVF (Fig 7A2), cultured (Fig 7A4) and fresh PN5 zygotes (Fig 7A6). Analysis of metaphase zygotes showed that culture from the early zygote stage caused variable levels of anti-5meC staining to persist in acid-only treated zygotes (Fig 7B). The level of methylation was assessed further by comparing staining with anti-MBD1 and anti-5meC in fresh and cultured zygotes (Fig 7C). This analysis showed that a similarly high level of MBD1 staining was observed in PN5 cultured (Fig 7C1) and fresh (Fig 7C3) zygotes, yet 5meC staining persisted in an asymmetrical fashion in cultured (Fig 7C7) but not fresh (Fig 7C9) zygotes. After acid and trypsin unmasking the MBD1 staining was lost from both treatments (Fig 7C2,4) and resulted in a similarly high level of staining with anti-5meC in both cultured and fresh zygotes (Fig 7C8,10). No staining was detected with non-immune control antisera for either antibody (Fig 7C5,6 and C11,12). The current results show that manipulation of the early embryo interferes with the maturational changes in zygotic chromatin that results in acid-resistant antigenic masking of 5meC, and this reduced level of masking was greatest in the female pronucleus giving an artifactual appearance of asymmetric demethylation.

These analyses were all undertaken on embryos from hybrid (B6CBF1) mice. To assess whether the strain of mouse influenced the patterns of anti-5meC staining we also examined staining in an inbred (C57BL/6j) and an outbred (Swiss Quackenbush) strain embryos. Zygotes were collected directly from the reproductive tract fixed and stained for anti-5meC and PI. Hybrid embryos were collected and processed at the same time to act as controls. This analysis showed in acid-treated zygotes there was a similar progressive loss of anti-5meC staining of both PN4 and PN5 pronuclei in all three strains (C57BL/6j Fig 8, panels 1 & 2; Quackenbush Fig 8, panels 5 & 6; Hybrid Fig 8, panels 9 & 10, respectively). In each strain trypsin treatment unmasked the 5meC antigen so that both pronuclei became heavily decorated with anti-5meC. It is concluded that the persistence of levels 5meC throughout maturation of the zygote is a normal feature of the development of the mouse embryo.

## Discussion

We used two different methods of analysis to reassess the widely accepted paradigm of extensive loss of cytosine methylation following fertilisation with further progressive demethylation over subsequent cell cycles. Given the small amount of DNA within the zygote this paradigm has been largely developed by use of immunolocalization of 5meC. The most recently published paper reporting this global demethylation [22] used this same approach but without the use of tryptic digestion for antigen retrieval. The current study shows that using previously published methods of immunolocalization of 5meC a major loss of staining occurred during zygotic maturation but this loss was not observed when nuclei were stained for the 5meC binding protein, MBD1. This difference in the results of the two staining methods was accounted for by the onset of a progressive acid resistant antigenic masking of 5meC. Tryptic digestion of zygotes removed this masking and revealed a persistence of 5meC during all stages of zygotic maturation and this staining persisted over subsequent cell-cycles.

The persistence of 5meC staining found in three different strains of mice, those conceived by IVF or in zygotes subjected to culture. No evidence for a marked asymmetry in methylation between the male and female pronuclei was detected after antigen retrieval with trypsin. Instead we found that when embryos are either produced by IVF or the zygotes were cultured from an early stage, the pattern of pronuclei maturation changed so that the extent of acid-resistant masking of 5meC was not as great in the female pronucleus. This differential in the extent of acid-resistant antigenic masking of 5meC that occurred after the manipulation of the embryo resulted in an asymmetry of anti-5meC staining. This gave the appearance of maintenance of the methylated state in the maternal pronucleus and its loss from the paternal pronucleus in cultured zygotes. Yet, this differential was not detected when MBD1 staining was used as a measure of global methylation levels, and the asymmetry was lost when full antigen retrieval was performed by tryptic digestion.

This study may reconcile some of the complexity in the field [23] that has resulted from reports of global demethylation in some mammalian species (mouse, rat, bovine [4]) but not others (sheep [9], [10], rabbit [11]). This discovery of a high degree of persistence of cytosine methylation after fertilisation may also explain why no mechanism capable of catalysing rapid global active demethylation has yet been discovered in mammals [7], [8] and is consistent with the discovery of the developmental viability of embryos where demethylation was not observed [14].

Immunolocalisation of 5meC or MBD1 provides a measure of the global levels of methylation in the embryo but clearly is not capable of informing us of the levels of methylation at individual loci or discrete regions of the genome. There is evidence for some level of remodelling of methylation at loci across the preimplantation stage of development. Most studies have been on very small numbers of loci [17], [24], [25], [26], making generalisation to the whole genome risky. A recent [2] wider scale analysis of the methylation of the genome of gametes showed that both egg and sperm have relatively low levels of methylation and that by the time the embryo had developed to the blastocyst stage this level was further reduced. This reduction, however, was not to a level predicted by the current model of epigenetic reprogramming and only a relatively small proportion of loci showed extensive demethylation [1]. The profound change in antigenic masking shown to occur during zygotic maturation, together with recent observations of a significant presence of 5-hydroxymethylcytosine within the mature zygotic pronuclei [27], and the presence and activity of DNMT1 in the early embryo [18] do not provide support for global loss of methylation as a consequence of fertilisation.

This study does not reveal the nature of the proteaceous masking of 5meC that occurs during zygotic maturation. 5meC acts as a docking site for a range of methylcytosine binding proteins and these in turn can recruit many other proteins to CpG islands. Changes in the identity, quantity or conformation of these proteins during zygotic maturation may mask the antigen. We show that MBD1 primarily co-localised with 5meC in the early embryo and that its removal by tryptic digestion was associated with the unmasking of the antigen. Extensive remodelling of histones within the zygotic genome also occurs during zygotic maturation [1], [15], [28], [29] and the resulting changes in the conformation of chromatin may have a role in the antigenic masking of 5meC. These profound changes in the conformation and structure of the zygotic genome coincides with the dramatic reprogramming of patterns of gene expression and the known transcription repression that occurs at this time [30]. Our observation that this occurs in the absence of profound changes in 5meC levels indicates that reprogramming of gene expression is not solely dependent upon demethylation. Our novel observation that IVF or culture of zygotes changed the level of antigenic masking, particularly in the female pronucleus reveals an unexpected perturbation of the maturation of zygotic chromatin structure/function as a consequence of IVF and zygote culture. Further analysis is required to establish what role this plays in the well described changes in the patterns of gene expression [31], [32] and long-term epigenetic programming [33], [34] that occur as a consequence of assisted reproductive technologies.

We observed changes in the pattern of 5meC immunolocalization during early development. The zygote to 2-cell transition was accompanied by a change from a generally diffuse pattern of staining across each pronuclei in the zygote to a pattern of more intense staining at the periphery of the nuclei of 2-cell and later stage embryos. In somatic cells it is recognised that much of the heavily methylated regions of the genome aggregate within heterochromatic regions of the genome. The localisation of heterochromatin varies between cell types and the status of the given cell, and it is not uncommon for heterochromatin to localise to the periphery of the nucleus. Little heterochromatin exits in the early zygote and it begins to form in the late zygote and 2-cell embryo [28]. It is likely that the change in the pattern of 5meC staining detected in the embryo reflects these maturational changes in nuclear architecture and further analysis of this point is required.

It is clear that the hypomethylated genome that the zygote inherits from gametes undergoes some CpG remodelling over the preimplantation period [3]. The current study shows that further global changes in the level of methylation in the immediate post-fertilisation period are not a major component of remodelling of the embryo's epi-genome. An understanding of the processes of epigenetic reprogramming in the early embryo requires more detailed whole genome analysis of ontogeny and mechanisms of CpG remodelling. The study also indicates that conclusions drawn from immunolocalization of 5meC in other cellular models may require reconsideration. Models of epigenetic reprogramming during development can be modified to exclude a genome-wide global progress of demethylation initiated by fertilisation.

## Materials and Methods

### Animals

The use of animals was in accordance with the Australian Code of Practice for the Care and Use of Animals for Scientific Purposes and was specifically approved by the Royal North Shore Hospital Animal Care and Ethics Committee (Protocol number 0711-044). Hybrid (C57BL/6j X CBA/He; B6CBF1) mice were used in most experiments. In the experiments reported in Figure 8, embryos were collected from inbred C57BL/6j and outbred Swiss Quackenbush strain females, each of which had been mated with males of the same strain. Animals were housed and bred in the Kearns Facility, Kolling Institute, St Leonards, NSW, Australia. All animals were under 12 h light: 12 h dark cycle and had access to food and water ad libitum. Six week old females were superovulated by intraperitoneal injection of 5 IU equine chorionic gonadotrophin (Folligon, Intervet International, Boxmeer, The Netherlands) followed 48 h later by 5 IU human chorionic gonadotrophin (hCG, Chorulon, Intervet). Females were paired with males of proven fertility. Pregnancy was confirmed by the presence of a copulation plug the following morning (day 1).

### Mouse embryo collection and culture

Embryos were collected from the reproductive tract in Hepes-buffered modified human tubal fluid medium (Hepes-mHTF) [35] at the times indicated in experiments. The timing of embryo collections was relative to the time of hCG administration ( h post-hCG). All components of the media were tissue culture grade (Sigma) and contained 3 mg bovine serum albumin per mL (CSL Ltd., Melbourne, Vic., Australia). In vitro fertilization (IVF) was performed as described [31]. Zygotes were collected from the oviduct at times shown in individual experiments. Earlier studies of zygote demethylation used simple culture systems. To try to recapitulate these conditions we cultured zygotes in modified human tubal fluid medium (mHTF) [35]. Zygotes were cultured individually in 10 µL volumes in 60-well culture plates (LUX 5260, Nunc, Naperville, IL) overlaid by approximately 2 mm of heavy paraffin oil (Sigma). Culture was at 37°C in 5% CO_2_ for the periods indicated in individual experiments. Embryos were treated with the DNA methyltransferases inhibitor N-Phthalyl-L-tryptophan (RG108, Sigma) [36] over the period of DNA replication in the 2-cell embryo [37].

### Immunolocalization and Western Analysis

Immunofluorescence of zygotes was performed as previously described [38]. Air-dried preparation of metaphase chromosome was by treating in 1% (w/v) sodium citrate solution for 3 min, and fixed in 3 parts methanol to 1 part acetic acid for 3 min, and dropped on an acid etched slide (modified from [16]). Interphase and some metaphase embryos were fix in 4% formaldehyde for 30 min. Fixed embryos were blocked in 30% serum overnight at 4°C and then incubated overnight at 4°C with primary antibodies: 5meC monoclonal staining was performed similarly to previous reports [1] with mouse anti-5meC antibody (1∶100 dilution, AbD Serotec, UK) and sheep anti-mouse IgG conjugated to fluorescein isothiocyanate (Sigma), or rabbit polyclonal to MBD1 (1∶100 dilution; Abcam, Cambridge, UK, ab3753) and goat anti-rabbit IgG (FITC-labelled, 1∶300 dilution; Sigma, St Louis or Alexa Fluor 633, red, 1∶500 dilution; Molecular Probes A21071 (when used in triple stain experiments)) DNA was counter stained with propidium iodide (PI) (Sigma, 0.1 µg/mL for interphase and 0.5 µg/mL for metaphase chromosomes) except where indicated. The primary observations of this study were confirmed on two separate conventional microscopes and two different confocal microscopes. The images shown in this work were performed by optical sectioning with a with a Leica TCS SP5 confocal microscope.

Unmasking of the 5meC antigen was performed by brief exposure to HCl as previously described [5]. In some experiments further unmasking was performed by tryptic digestion (0.25% (w/v) trypsin at 37°C for 45 sec, Invitrogen, Carlsbad, CA). Digestion was stopped by washing in 10% (v/v) serum.

Embryos from each treatment were processed at the same time and in parallel for each experimental replicate. All treatments were exposed to the same preparations and dilutions of all reagents including primary and secondary antibodies. Similarly all preparations from an experiment were examined microscopically within the same session, and used identical microscope and camera settings. All image analysis was performed in an identical manner for all embryos within an experiment. All preparations were performed by the same experienced operator throughout the study. Semi-quantitative analysis of staining was independently confirmed by two experienced observers.

Western blot analysis on embryo and oocyte extracts was performed as previously described [39] using rabbit anti-MBD1 (1∶500, Abcam). This was detected with goat-anti-Rabbit HRP conjugated antibodies (1∶5000). Each lane had the protein from 50 embryos or oocytes. The two bands of ∼55 and 134 kDa were found at each stage of development. The blot was stripped and re-probed with antibody directed against beta-actin and the optical density (OD) of the MBD1 bands is shown relative to the beta-actin band.
